# Monoclonal Antibodies to the V2 Domain of MN-rgp120: Fine Mapping of Epitopes and Inhibition of α4β7 Binding

**DOI:** 10.1371/journal.pone.0039045

**Published:** 2012-06-13

**Authors:** Gerald R. Nakamura, Dora P. A. J. Fonseca, Sara M. O'Rourke, Aaron L. Vollrath, Phillip W. Berman

**Affiliations:** 1 Antibody Engineering Department, Genentech, Incorporated, South San Francisco, California, United States of America; 2 Department of Biomolecular Engineering, University of California Santa Cruz, Santa Cruz, California, United States of America; University of California San Francisco, United States of America

## Abstract

**Background:**

Recombinant gp120 (MN-rgp120) was a major component of the AIDSVAX B/E vaccine used in the RV144 trial. This was the first clinical trial to show that vaccination could prevent HIV infection in humans. A recent RV144 correlates of protection study found that protection correlated with the presence of antibodies to the V2 domain. It has been proposed that antibodies to the α4β7 binding site in the V2 domain might prevent HIV-1 infection by blocking the ability of virions to recognize α4β7 on activated T-cells. In this study we investigated the specificity of monoclonal antibodies (MAbs) to the V2 domain of MN-rgp120 and examined the possibility that these antibodies could inhibit the binding of MN-rgp120 to the α4β7 integrin.

**Methodology/Principal Findings:**

Nine MAbs to the V2 domain were isolated from mice immunized with recombinant envelope proteins. The ability of these MAbs to inhibit HIV infection, block the binding of gp120 to CD4, and block the binding of MN-rgp120 to the α4β7 integrin was measured. Mutational analysis showed that eight of the MAbs recognized two immunodominant clusters of amino acids (166–168 and 178–183) located at either end of the C strand within the four-strand anti-parallel sheet structure comprising the V1/V2 domain.

**Conclusions/Significance:**

These studies showed that the antigenic structure of the V2 domain is exceedingly complex and that MAbs isolated from mice immunized with MN-rgp120 exhibited a high level of strain specificity compared to MAbs to the V2 domain isolated from HIV-infected humans. We found that immunization with MN-rgp120 readily elicits antibodies to the V2 domain and some of these were able to block the binding of MN-rgp120 to the α4β7 integrin.

## Introduction

Recombinant gp120 from the MN strain of HIV-1 (MN-rgp120) has been investigated as a candidate HIV-1 vaccine to elicit protective antibody responses [1]–[4]. Two bivalent HIV-1 subunit vaccines, AIDSVAX B/B and AIDSVAX B/E, each containing MN-rgp120, have been developed for use in North America and Thailand, respectively [5], [6]. Both vaccines have now been tested in large scale Phase 3 trials alone, or in combination with vaccines such as vCP1521 designed to stimulate cellular immune responses [7]. In the VAX003 and VAX004 trials (1998–2003), immunization with these vaccines was ineffective in preventing new HIV-1 infections in cohorts of injection drug users (IDUs) and men who had sex with men (MSMs) [8], [9]. However, the RV144 clinical trial showed that a prime/boost immunization regimen, involving priming immunizations with a recombinant canarypox vector vCP1521 followed by booster immunizations with AIDSVAX B/E, provided modest but significant protection from heterosexual HIV-1 transmission [7]. This trial provided the first evidence that vaccination can prevent HIV-1 infection in humans. In order to establish a correlate of protection, there is renewed interest in defining the specificity of the antibody response to the vaccine immunogens, including MN-rgp120. Initial analysis of sera using the TZM-bl virus neutralization assay [10] failed to document a correlation between the level of virus neutralizing antibodies and protection [11], [12]. As a consequence, multiple investigators have begun to consider the possibility that antibodies might confer protection *in vivo* by means other than direct virus neutralization. In principle, non-neutralizing antibodies might confer protection by effectively lowering the probability of infection from transmitted virus inocula. Several distinct mechanisms have been proposed by which non-neutralizing antibodies to the HIV-1 envelope protein might have a protective effect. These include inactivation of viruses or virus-infected cells by antibody-dependent cell-mediated virus inhibition [13]; aggregation of virions at mucosal surfaces, thus impairing virus transport across mucosal membranes [14]; or prevention of viruses from selectively targeting activated CD4^+^ T-cells [15].

Recently, antibodies that bind to the V2 domain of the HIV-1 envelope protein gp120 have attracted considerable attention in connection with the RV144 trial [16]. The V2 domain is known to play a major role in conformational masking, shielding epitopes located in other regions of the molecule from exposure to virus neutralizing antibodies [17]–[19]. Two recent studies have reported that potent neutralizing antibodies in sera from HIV-1 infected individuals are directed to the V2 domain [20], [21]. Moreover, two extremely potent broadly neutralizing monoclonal antibodies (MAbs), PG9 and PG16, have been shown to target an epitope in the V2 domain [22]. Finally, it has been demonstrated that the V2 domain of gp120 can serve as a ligand for the T-cell associated integrin, α4β7 [23]. It has been proposed that α4β7 interactions play a major role in facilitating HIV-1 infection by enabling HIV-1 virions to target activated CD4+ T-cells. It has been known for many years that infection of activated CD4+ cells results in productive HIV-1 infection, whereas infection of un-activated CD4+ cells is less efficient and typically results in abortive infection [24]–[26]. However, until the discovery of the α4β7 receptor binding site in the V2 domain of gp120, it was not known how HIV-1 could bypass the vast majority of un-activated CD4+ cells in humoral circulation and target the few activated CD4+ cells that could sustain productive infections. The discovery of the α4β7 binding site in gp120 provides a plausible explanation for this important aspect of HIV-1 biology [15]. Thus, antibodies to the V2 domain that inhibit the binding of HIV-1 to α4β7 would be expected to lower the probability of infection and might provide an explanation for the modest protective effect seen in the RV144 trial.

## Results

### Monoclonal antibodies to the V2 region of MN-rgp120

**Figure 1:**
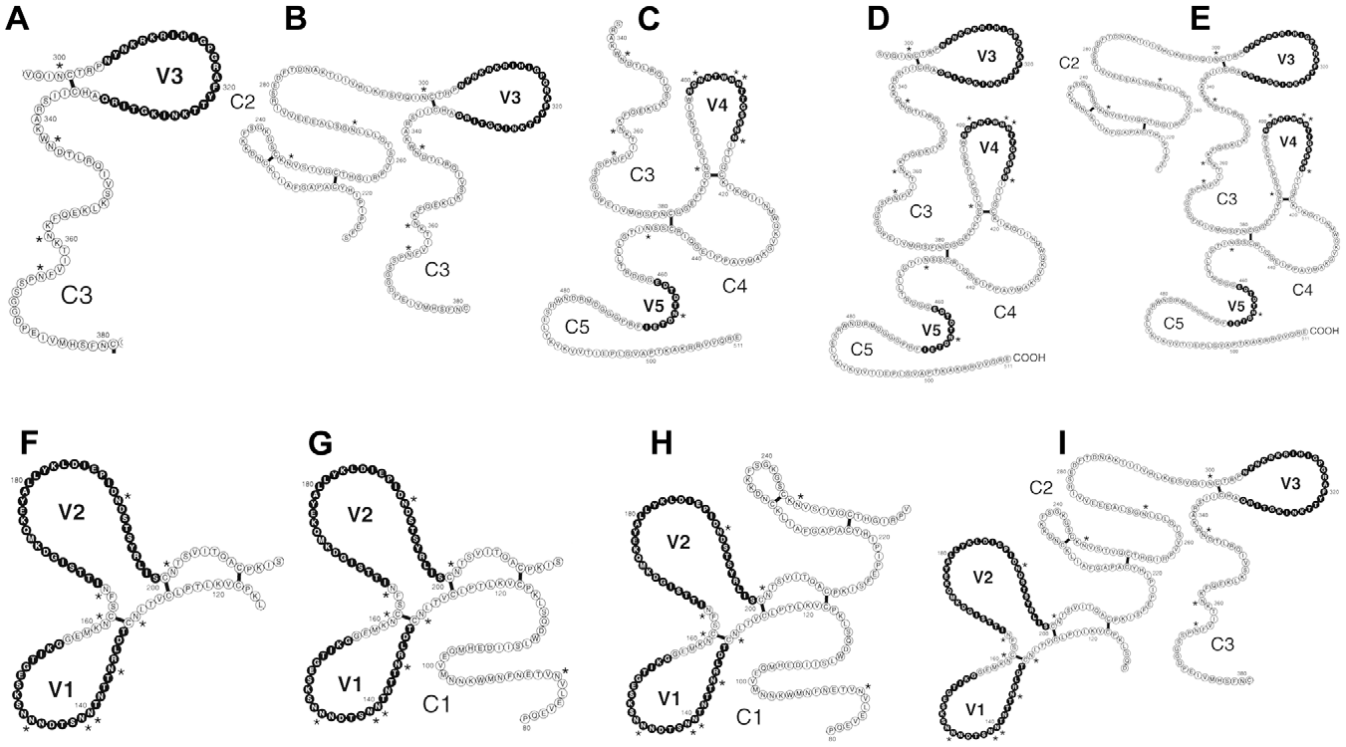
Fragments of gp120 used for epitope mapping studies. Fragments of the gp120 gene were selected based on the two-dimensional structure of Leonard et al. [54] and fused to the signal sequence and 27 N terminal amino acids of glycoprotein D from type 1 herpes simplex virus. The fragments were expressed in 293 cells, and cell culture supernatants containing the secreted fragments were screened by ELISA for MAb binding. MAb binding to fragments A–E localized epitopes outside of the V1 and V2 domains; MAb binding to fragments F–I localized epitopes that depended on the V1 and V2 domain.

A collection of thirty-six monoclonal antibodies, produced in the mid-1990's against MN-rgp120 or IIIB-rgp140 [27], [28], were screened for reactivity to the MN-rgp120 V2 domain. For this purpose, a novel epitope mapping strategy was employed using a panel of overlapping fragments of MN-rgp120, expressed in mammalian cells (Figure 1, A–I). As described previously [28], fragments of gp120 fused to the N-terminus of herpes simplex virus type 1 glycoprotein D (gD-1), possessing the signal sequence and a 27 amino acid flag epitope, can be efficiently secreted from 293 cells. Moreover, they often preserve some structural features such as N-linked glycosylation, disulfide bonds, and epitopes recognized by conformation-dependent antibodies found on full length gp120. Initial binding studies showed that nine of the thirty-six MAbs bound to MN-rgp120 fragments F–I containing the V1 and V2 domains (Table 1; Figure 1). The shortest fragment (F) extended from amino acid position 115 to 211, and included the V1/V2 stem and the entire V1 and V2 loops. MAbs 1025 and 6E10 (isolated from a mouse immunized with IIIB-rgp140) failed to bind to the V1/V2 fragment but only recognized the largest (fragment I) that included the V1 to C3 domains (residues 104 to 371). Isotype analysis showed that all of the MAbs were of the IgG1 isotype, with the exception of 1022, which was IgG2a, and 1029, which was IgG2b.

**Table 1:** Properties of V2 region monoclonal antibodies.

MAbs	Isotype	RCM-rgp120	HIV rgp120 Binding	CD4 Blocking	Neutralization (µg/mL)	Epitope Localization
1019	IgG1	+/−	MN	+	23	V1, V2
1027	IgG1	+/−	MN	+	17	V1, V2
1017	IgG1	−	MN	+	23	V1, V2
1029	IgG2b	−	MN, IIIB	+	>25	V1, V2
1022	IgG2a	−	MN	−	>25	V1, V2
1028	IgG1	−	MN, IIIB	+	>25	V1, V2
1088	IgG1	+++	MN	++	0.07	V1, V2
1025	IgG1	−	MN	+	6.25	V1, V2, C2, V3, C3
6E10*	IgG1	−	MN, IIIB, JRCSF	+	2.0	V1, V2, C2, V3, C3
*1026*	IgG1	ND	MN, IIIB, JRCSF	−	0.01	V3
*b12*	IgG1	ND	ND	++	0.02	C2, C3, C4, C5
*PG9*	IgG1	ND	ND	−	>25	V2

RCM-rgp120, indicates the ability of MAbs to bind to denatured (reduced and carboxymethylated) gp120. HIV rgp120 binding, indicates the ability of MAbs to bind to recombinant gp120 from the MN, IIIB and JRCSF strains of HIV in an ELISA format. CD4 blocking represents a summary of the data provided in Supplemental Figure S1. Neutralization data represents the MAb concentration (µg/mL) required for 50% inhibition (IC50) of infectivity by the MN-3 strain of HIV-1 in the TZM-bl neutralization assay (Materials and Methods) with the exception of the 6E10 MAb (*) where neutralization titers were measured using the IIIB strain of gp120 as described previously [27]. Controls for the assays (italic) included MAbs 1026, b12 and PG9. ND, indicates not done. Epitope localization, summarizes the minimal gp120 structure required for antibody binding based on MAb reactivity with the gp120 fragments shown in Figure 1.

To determine whether the V2 MAbs recognized linear or conformation-dependent epitopes, MAb binding to unfolded, reduced and carboxymethylated MN-rgp120 (RCM-rgp120) was examined. It was found (Table 1) that six of the nine MAbs tested (6E10, 1017, 1022, 1025, 1028, and 1029) had no reactivity with RCM-rgp120; two MAbs, 1019 and 1027, gave weak binding to RCM-rgp120; and one MAb (1088) exhibited strong reactivity with the denatured rgp120. These results demonstrated that the immunization and screening procedure employed favored the isolation of MAbs that recognized conformation-dependent epitopes.

Further information regarding the specificity of the V2 MAbs was obtained in rgp120 cross-reactivity studies. In these experiments, the MAb binding to recombinant gp120 from seven diverse isolates of HIV-1 (MN, IIIB, NY5, JRCSF, A244, Z321, and Z6) was measured. The results obtained (Table 1) indicated the cross-reactivity was extremely limited, with six of the MAbs (1017, 1019, 1022, 1025, 1027, and 1088) reacting exclusively with MN-rgp120. MAbs 1028 and 1029 bound both MN-rgp120 and IIIB-rgp120, whereas the 6E10 MAb bound the MN, IIIB, and JRCSF derived proteins. In control experiments (data not shown), MAb 1034, directed to the MN V3 domain, bound rgp120 from the MN, IIIB, NY5, JRCSF, and Z321 isolates. These results demonstrated that the V2-directed MAbs exhibited a high degree of strain specificity.

### *In vitro* neutralization activity

We found (Table 1) that six of the nine MAbs demonstrated virus neutralizing activity. Of these, three (1019, 1027, and 1017) had weak neutralizing activity (IC50≥17 µg/mL) against the MN-3 strain of HIV-1. Two (1088, 1025) had potent neutralizing activity against the MN-3 strain (IC50 of 0.07 and 6.25 µg/mL, respectively). Finally, the 6E10 MAb neutralized only the IIIB strain of HIV-1 with an IC50 of 2.0 µg/mL [27]. Of these, the 1088 MAb was the most potent, with an IC50 in the same order of magnitude as the positive control antibodies directed to the V3 domain (1026) and the CD4 binding site (b12). The prototypic broadly neutralizing antibody to the V2 domain, PG9, had no neutralizing activity in this assay and was unable to bind to purified MN-rgp120 by ELISA (data not shown). Interestingly the 1088 antibody was the only V2 MAb that exhibited strong reactivity with denatured (reduced and carboxymethylated) gp120 indicating that it recognizes a conformation-independent epitope.

### Inhibition of rgp120 binding to CD4 by V2 MAbs

The ability of the nine V2 directed MAbs to inhibit binding of [^125^I]-MN-rgp120 to cell surface CD4 (Supplemental Figure S1) was measured in assays similar to those described previously [28]. MAb 1088 completely inhibited the binding of MN-rgp120 to CD4 with an IC50 of 1.0 µg/mL, whereas MAbs 1017, 1019, 1025, 1027, 1028, 6E10, and 1029 exhibited partial (i.e., 30%) inhibition at a concentration of 10.0 µg/mL and gave no more than 50% inhibition even at antibody concentrations exceeding 100 µg/mL. The CD4-blocking activity was apparent for the 1088 MAb in cell surface binding assays and in ELISA assays; however, the CD4-blocking activity by the other MAbs was apparent only in the cell surface binding assay. These data suggest that MAbs to the V1/V2 region of gp120 can alter the binding of rgp120 to CD4, and that some MAbs (e.g., 1088) have potent CD4 blocking activity and potent neutralizing activity.

### Cross-competition studies

**Figure 2:**
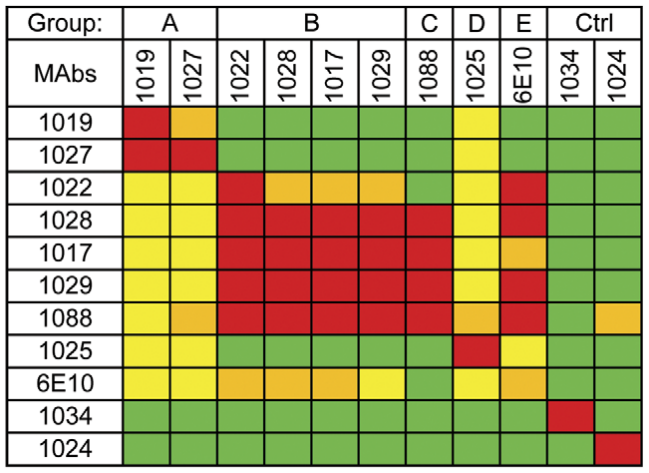
Competitive binding of monoclonal antibodies. Each monoclonal antibody was labeled with horseradish peroxidase (HRPO). The ability of a large excess of each unlabeled antibody to inhibit the binding of HRPO MAbs was measured. Inhibition of greater than 50% was considered significant and was defined as directly competitive. Isotype matched antibodies to the V3 domain (1034) and the C4 domain (1024) served as negative controls. Data represent percentage of inhibition: red, 90–100%; orange, 70–89%; yellow, 50–69%; green, <50%.

Competitive binding assays (Figure 2) provided data on the specificity of the different MAbs. In these studies, unconjugated MAbs were allowed to react with rgp120-coated microtiter dishes followed by a second incubation with horseradish peroxidase (HRPO) labeled V2 MAbs. Inhibition of greater than 50% was considered significant and was defined as directly competitive. Based on the competitive binding data (Figure 2), the MAbs could be divided into five distinct competition groups (A, B, C, D, and E). Group A consisted of MAbs 1019 and 1027, whereas group B included antibodies 1017, 1022, 1028, and 1029. MAbs 1088, 1025, and 6E10 represented distinct groups designated C, D, and E, respectively. MAbs 1019, 1027, and 1025 were notable in that they competed for binding with all of the V2 region MAbs described in this paper.

### Epitope mapping by site-directed mutagenesis of the V2 domain

**Figure 3:**
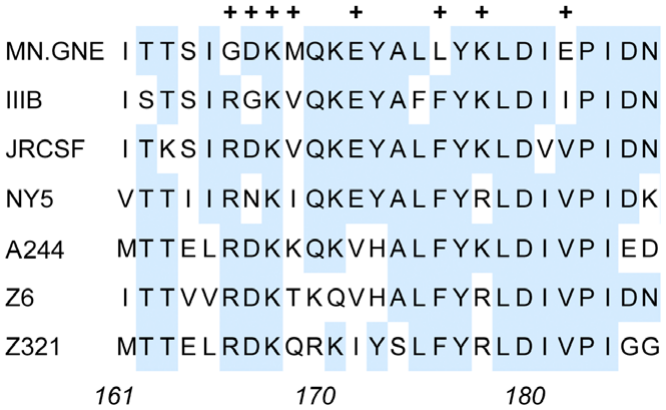
Alignment of V2 domains of HIV-1 envelope proteins that differed in binding V2 MAbs. Sequences from the envelope proteins used in ELISA assays to detect the binding of V2 MAbs were aligned. Homologous sequences were shaded in blue. Plus (+), indicates location of sites selected for mutagenesis. Numbering is provided with reference to the sequence of the HXB2 envelope protein.

We next characterized the epitopes recognized by these MAbs. Because most of the MAbs exhibited weak binding to a MN-rgp120 fragment consisting of only the V2 domain (data not shown), we considered the possibility that the epitopes were contained entirely in this region. Our initial strategy depended on site-directed mutagenesis, where naturally occurring sequence polymorphisms were incorporated into MN-rgp120 based on comparisons of the sequence of MN-rgp120 with envelope proteins from strains (Figure 3) that failed to bind the V2 MAbs. One advantage of this strategy is that it relied on naturally occurring mutations used by the virus to escape the immune response, and avoided unphysiologic substitutions that were more likely to disrupt the structure of the V2 domain. In the first series of MN-rgp120 variants, we substituted residues from HIV-1 envelope proteins unable to bind the MAbs into the MN-rgp120 sequence. The panel consisted of proteins with the following replacements: G166R, M169V, L176F, E182V, and P183S (Table 2). A second series of experiments consisted of single and double alanine (A) mutations where charged residues were replaced by the non-polar alanine residue. These included D167A-K168A, E172A, K178A, and E182A. In control experiments, variants of MN-rgp120 containing mutations in regions outside the V2 domain that were described previously [27], were also assayed (data not shown). The results of these studies identified six different antibody specificities.

**Table 2:** Listing of V2 region mutations incorporated into MN-rgp120.

	*161*								*170*										*180*							
MN	I	T	T	S	I	G	D	K	M	Q	K	E	Y	A	L	L	Y	K	L	D	I	E	P	I	D	N
G166R	-	-	-	-	-	R	-	-	-	-	-	-	-	-	-	-	-	-	-	-	-	-	-	-	-	-
G166R, E182V, P183S	-	-	-	-	-	R	-	-	-	-	-	-	-	-	-	-	-	-	-	-	-	V	S	-	-	-
G166R, M169V	-	-	-	-	-	R	-	-	V	-	-	-	-	-	-	-	-	-	-	-	-	-	-	-	-	-
G166R, M169V, E182V, P183S	-	-	-	-	-	R	-	-	V	-	-	-	-	-	-	-	-	-	-	-	-	V	S	-	-	-
D167A, K168A	-	-	-	-	-	-	A	A	-	-	-	-	-	-	-	-	-	-	-	-	-	-	-	-	-	-
M169V	-	-	-	-	-	-	-	-	V	-	-	-	-	-	-	-	-	-	-	-	-	-	-	-	-	-
E172A	-	-	-	-	-	-	-	-	-	-	-	A	-	-	-	-	-	-	-	-	-	-	-	-	-	-
L176F	-	-	-	-	-	-	-	-	-	-	-	-	-	-	-	F	-	-	-	-	-	-	-	-	-	-
K178A	-	-	-	-	-	-	-	-	-	-	-	-	-	-	-	-	-	A	-	-	-	-	-	-	-	-
E182A	-	-	-	-	-	-	-	-	-	-	-	-	-	-	-	-	-	-	-	-	-	A	-	-	-	-
E182V	-	-	-	-	-	-	-	-	-	-	-	-	-	-	-	-	-	-	-	-	-	V	-	-	-	-
P183S	-	-	-	-	-	-	-	-	-	-	-	-	-	-	-	-	-	-	-	-	-	-	S	-	-	-
E182V, P183S	-	-	-	-	-	-	-	-	-	-	-	-	-	-	-	-	-	-	-	-	-	V	S	-	-	-
E182V, P183V	-	-	-	-	-	-	-	-	-	-	-	-	-	-	-	-	-	-	-	-	-	V	V	-	-	-

**Figure 4:**
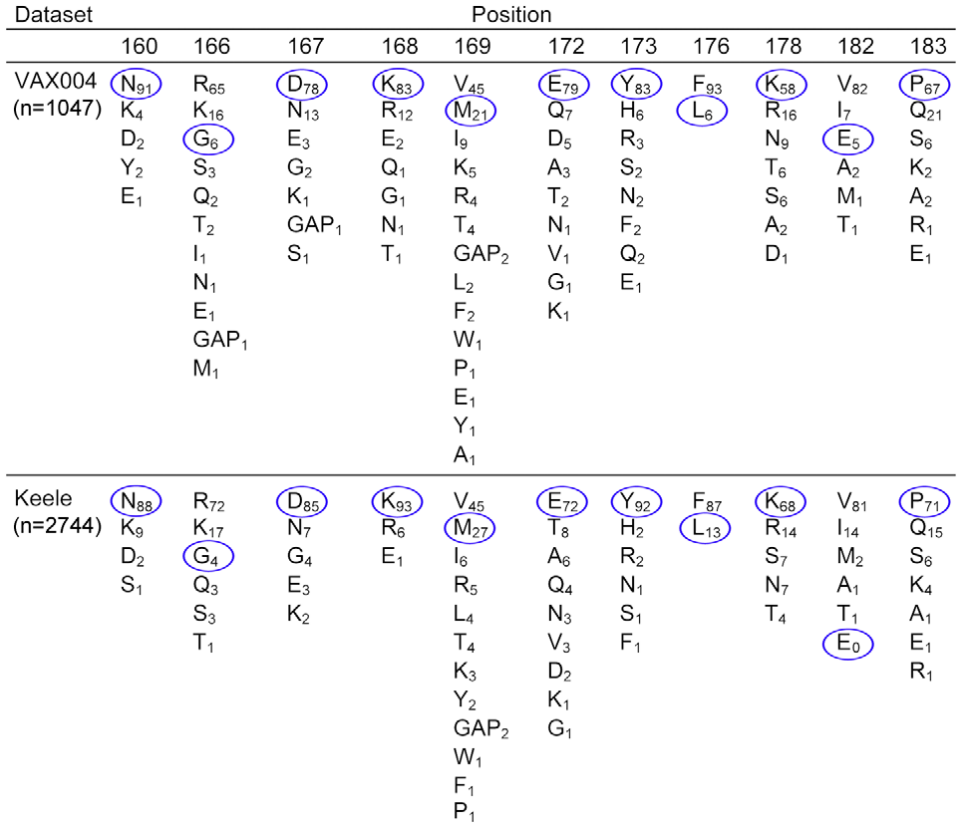
Frequency of sequence polymorphisms at positions in the V2 domain recognized by monoclonal antibodies to MN-rgp120. Letters represent standard single letter amino acid codes. GAP indicates the frequency of a deletion at a given position. Subscripts represent the percentage of sequence polymorphism at a single position in each dataset. Positions are numbered according to the HXB2 reference sequence. The VAX004 dataset includes 1047 clade B sequences from 349 individuals. The Keele data set represents 2744 clade B sequences from 102 individuals. Residues circled in blue indicate amino acids present in the MN_GNE_ strain of HIV-1. VAX004 data obtained from Pérez-Losada et al., 2010 [60], using data listed in the GSID HIV Data Browser http://www.gsid.org/gsid_hiv_data_browser.html. Sequences of clade B envelope proteins from new infections were obtained from Keele et al. [29] using data listed in the Los Alamos HIV Sequence Database http://www.hiv.lanl.gov/content/sequence/HIV/mainpage.html.

The binding of the competition group A antibodies, 1019 and 1027, could be inhibited by the naturally occurring G166R mutation and the double Ala mutation of D167A- K168A (Table 3). These data did not allow us to know whether the D167A-K168A mutations, alone or in combination, are responsible for inhibiting the binding of these MAbs. We found that the binding of these MAbs was unaffected by the M169V and E172A mutations which are in close proximity to positions 166–168. Based on these data, our results allowed us to define an epitope termed MN.V2.1 that involves residues G166, D167, and/or K168 (Table 4). Examination of the consensus sequence for Clade B gp120s from the VAX004 trial (1024 sequences) and the Keele dataset (2744 sequences) of viruses from new infections [29] (Figure 4) suggests that G at position 166 is rare and occurs only at 4–6% of clade B isolates, whereas R at this position occurs in 65–72% of clade B viruses. This mutation may explain the lack of cross-reactivity and cross-neutralizing activity of these MAbs with envelopes from other strains of HIV-1.

**Table 3:** Relative binding of V2 MAbs to MN-rgp120 V2 mutants.

Group A		Group B				Group C	Group D	Group E	Control	Mutations
1019	1027	1022	1028	1017	1029	1088	1025	6E10	1034	
0.00	0.00	0.60	0.62	0.64	0.63	0.63	0.53	0.03	0.68	G166R
0.00	0.00	0.00	0.01	0.09	0.11	0.00	0.67	0.00	0.87	G166R, E182V, P183S
0.21	0.00	0.88	0.86	0.88	0.85	0.83	0.79	0.55	0.86	G166R, M169V
0.12	0.00	0.00	0.07	0.21	0.17	0.01	0.89	0.69	0.99	G166R, M169V, E182V, P183S
0.00	0.00	1.24	1.26	1.20	1.14	1.19	1.13	1.15	1.21	D167A, K168A
1.17	1.05	1.12	1.21	1.23	1.21	1.15	1.10	1.73	1.15	M169V
1.91	2.13	1.68	1.57	1.49	1.40	1.53	0.13	2.25	1.30	E172A
0.96	1.00	1.13	1.03	1.08	1.02	1.03	0.05	0.94	0.89	L176F
1.06	1.09	0.00	0.00	0.00	0.05	0.00	1.05	0.00	1.04	K178A
0.96	1.11	1.16	1.17	1.15	1.10	1.09	1.05	1.01	1.06	E182A
0.75	1.26	0.24	0.06	1.23	0.72	0.78	1.17	1.45	0.98	E182V
0.67	1.04	0.00	0.24	0.15	0.28	0.00	0.87	0.28	1.00	P183S
0.34	0.45	0.00	0.06	0.13	0.15	0.01	0.71	0.01	0.72	E182V, P183S
0.07	0.14	0.00	0.20	0.35	0.19	0.08	0.34	0.00	0.61	E182V, P183V

Data represent ratios of ELISA results where MAb binding to envelope proteins with the mutations indicated was compared to binding of the wildtype MN-rgp120. Significant differences in MAb binding are represented by underlined numbers. Percentage inhibition is calculated according to the following equation: (1 – binding to mutant/binding to MN-rgp120)×100.

**Table 4:** Summary of V2 epitopes recognized by Murine MAbs to MN-rgp120.

MAbs	Competition Group	Contact Residues	Epitope
1019	A	G166, D167, K168	MN.V2.1
1027	A	G166, D167, K168	MN.V2.1
1017	B	K178, P183	MN.V2.2
1029	B	K178, P183	MN.V2.2
1022	B	K178, E182, P183	MN.V2.3
1028	B	K178, E182, P183	MN.V2.3
1088	C	K178, P183	MN.V2.4
1025	D	E172, L176	MN.V2.5
6E10	E	G166, K178, P183	MN.V2.6

We next examined the competition group B MAbs (Table 3). We found that the K178A and P183S mutations, independently, inhibited the binding of all four of the group B MAbs (1022, 1028, 1017, and 1029). Therefore these two mutations define a second epitope, termed MN.V2.2, that is at least ten amino acids away from the MN.V2.1 epitope (Table 4). Although both positions in the MN.V2.2 epitope are polymorphic, K at position 178 and P at position 183 are the most common amino acids at these positions among clade B viruses (Figure 4). Further analysis showed that competition group B included a second epitope, termed MN.V2.3, that was defined by the radical E182V mutation present in the MN envelope protein, but few (0–5%) other clade B envelopes (Table 4 and Figure 4). This mutation inhibited the binding of MAbs 1022 and 1028 by 76% and 94%, respectively, whereas the binding of the other competition group B MAbs was not inhibited by the E182V mutation. The 1028 MAb in competition group B was unique in that it cross-reacted with IIIB-rgp120, but the basis for this differential recognition is not yet known. Thus the MN.V2.3 epitope appears to be a discontinuous epitope, where additional contact residues shared between MN- and IIIB-rgp120s are required for the binding of this MAb (Table 4).

Competition group C is represented by MAb 1088. This MAb was unique in that it recognized a conformation-independent epitope, as indicated by its ability to bind RCM-rgp120, and by the fact that it exhibited potent, type-specific, neutralizing activity (Table 1). In addition, this MAb had the most potent CD4-blocking activity (IC50 = 1.0 µg/ml) of any of the V2-specific MAbs isolated. Like the MN.V2.2 and MN.V2.3 epitopes, binding by this antibody was independently inhibited by the K178A and P183S mutations, but was unaffected by the E182V mutation. Based on its binding to denatured gp120, its competition pattern, and its biologic activities, this antibody clearly represents a fourth epitope, MN.V2.4 (Table 4). Interestingly, the K178A, E182V, and P183S mutations occur immediately adjacent to the highly conserved α4β7 recognition sequon, LDI/V, spanning positions 179–181 [23]. Therefore these MAbs appear to have the potential to inhibit the binding of gp120 to the α4β7 receptor.

The competition group D MAb, 1025, resembled the competition group A antibodies in that it competed for binding with all of the V2 MAbs in this study (Figure 2). However, it differed from the competition group A antibodies in that it required the C2, V3, and C3 domains, as well as the V1 and V2 domains, and it exhibited modest virus neutralizing activity. Mutational analysis showed that two mutations, E172A and L176F, uniquely resulted in an 87% and 95% reduction, respectively, in binding of this MAb. These results demonstrated that the 1025 MAb recognized a distinct new epitope, MN.V2.5 (Table 4). Examination of the HIV-1 sequence database revealed that both positions were polymorphic, with V or E most commonly occurring at position 172 and F most commonly occurring at position 176 (Figure 4). The 1025 MAb was also unique in that it was the only MAb that was insensitive to the double mutant E182V-P183S, as well as the triple mutant G166R- E182V-P183S. Thus this epitope is clearly different from the other epitopes described and seems to recognize a site located between the MN.V2.1 and MN.V2.2 epitopes.

The competition group E antibody 6E10, which was isolated from a mouse immunized with IIIB-rgp140 [27], cross-reacted with rgp120 from the IIIB, MN and JRCSF isolates, and neutralized the homologous IIIB isolate. Like the competition group A MAbs, the binding of this antibody was inhibited independently by the G166R mutation. However, like the competition group B mutants, the binding of 6E10 was also inhibited by the K178A mutation. These data suggest that the 6E10 MAb reacts with a distinct epitope termed MN.V2.6 that overlaps the binding sites of the group A and group B epitopes (Tables 3 and 4). Like the 1025 MAb, this antibody appeared to recognize a conformation-dependent, discontinuous epitope that required the C2, V3, and C3 domains for binding as well as the V1 and V2 domains.

Finally, we examined the effect of combining multiple mutations. We observed that several double and triple mutants appeared to prevent the binding of most of the V2 antibodies in the panel. We found that the E182V-P183S mutant and the E182V-P183V mutant prevented the binding of all of the MAbs in the panel except 1025, whereas other double mutants such as G166R-M169V and D167A-K168A selectively inhibited MAb binding. This result suggested that positions 182 and 183 are unique in that they appear to be critically important for maintaining a unique conformation or structural feature important for the binding of all of the MAbs in this study except for 1025. In view of this observation, it is not surprising that the triple mutant G166R-E182V-P183S similarly prevented the binding of eight of the nine MAbs in this study. As noted above, positions 182 and 183 occur immediately adjacent to the α4β7 recognition sequence LDI spanning position 179–181.

### Inhibition of gp120 binding to α4β7

**Figure 5:**
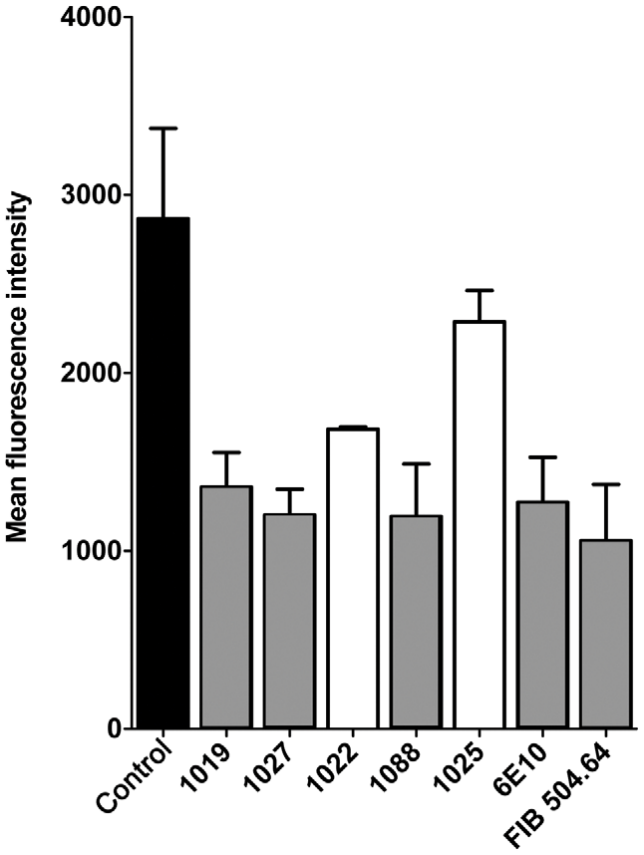
Inhibition of MN-rgp120 binding to α4β7 by monoclonal antibodies to the V2 domain. Biotinylated MN-rgp120 was incubated with monoclonal antibodies to the V2 domain of gp120 for 1 hr at room temperature. Antibodies representative of the five V2 competition groups were tested: Group A, 1019 and 1027; Group B, 1022; Group C, 1088; Group D, 1025; Group E, 6E10. The mixture was then added to cells and incubated for 1 hr at 4°C. After washing, the cells were incubated with APC-conjugated streptavidin to detect gp120 binding. The amount of gp120 binding to cells was measured by flow cytometry and statistical analysis was carried out by one way ANOVA including a Bonferroni correction. The binding of MN-rgp120 to α4β7 without added MAbs (control) is indicated by the black bar. Statistically significant inhibition of MN-rgp120 binding (p<0.05) is indicated by gray bars; inhibition that does not reach statistical significance is indicated by white bars. Inhibition of MN-rgp120 binding by the FIB504.64 MAb to α4β7 was measured as described in Materials and Methods.

We next investigated the potential of the MAbs to the V2 domain to block the binding of MN-rgp120 to the α4β7 receptor. For this purpose we selected the RPMI-8866 B cell lymphoma cell line [30], [31] that is known to express α4β7, but does not express CD4, the receptor for HIV-1. By using a cell line lacking CD4, we avoided the necessity of using anti-CD4 antibodies to block gp120 binding to CD4 as has been described in previous studies where the binding of gp120 to α4β7 was measured [23]. The binding of MN-rgp120 to α4β7 was carried out with the use of biotinylated MN-rgp120 and detected by flow cytometry using APC-conjugated streptavidin. In preliminary studies, we measured the binding of MN-rgp120 to the RPMI-8866 cells and demonstrated the specificity of this binding by showing that binding could be inhibited by antibodies to α4β7 or by lowering the concentration of Ca^2+^ and Mn^2+^ (data not shown) which are essential ions for the binding of α4β7 to its normal receptor, MAdCAM. In these experiments we screened several MAbs raised against α4β7 (see Materials and Methods), and found that the FIB504.64 MAb was particularly effective in blocking MN-rgp120 binding to α4β7. We then examined the ability of MAbs to the V2 domain to inhibit binding to α4β7. For these studies, we utilized antibodies from all five competition groups. We found (Figure 5) that antibodies from competition groups A, C, and E significantly (p<0.05) inhibited MN-rgp120 binding to α4β7. Interestingly, the A, C, and E groups all depended on positions 178 and 183 which flanked the α4β7 binding site (LDI) located at positions 179, 180, and 181. In contrast, the group D MAb, 1025, whose binding is dependent on positions 172 and 176, was ineffective in blocking binding to α4β7. We cannot explain the inability of the group B MAb 1022 to block α4β7 binding, since it also recognizes positions 178, 182, and 183. Thus, antibodies from three of five competition groups were able to block the binding of MN-rgp120 to α4β7 binding in this assay, and the 1027, 1088, and 6E10 MAbs were as effective as the FIB504.64 antibody in blocking binding to α4β7.

## Discussion

In the present study, we used a novel strategy to characterize the binding specificity of nine MAbs reactive with the V2 domain of MN-rgp120. Initial localization of epitopes was carried out with a series of overlapping, glycosylated fragments of gp120 expressed in mammalian cells. Epitope fine mapping was determined by site-directed mutagenesis where the impact of naturally occurring sequence polymorphisms on antibody binding to the V2 domain was measured. In addition, we describe a novel assay using cells from the RPMI-8866 B-cell lymphoma to measure the binding of gp120 to the α4β7 integrin and the ability of antibodies to the V2 domain of gp120 to inhibit this binding. Because the RPMI-8866 cells do not express CD4, it eliminates the need to block the high affinity binding of gp120 to CD4 that was a significant complication in previous studies of gp120 binding to α4β7 [23].

**Figure 6:**
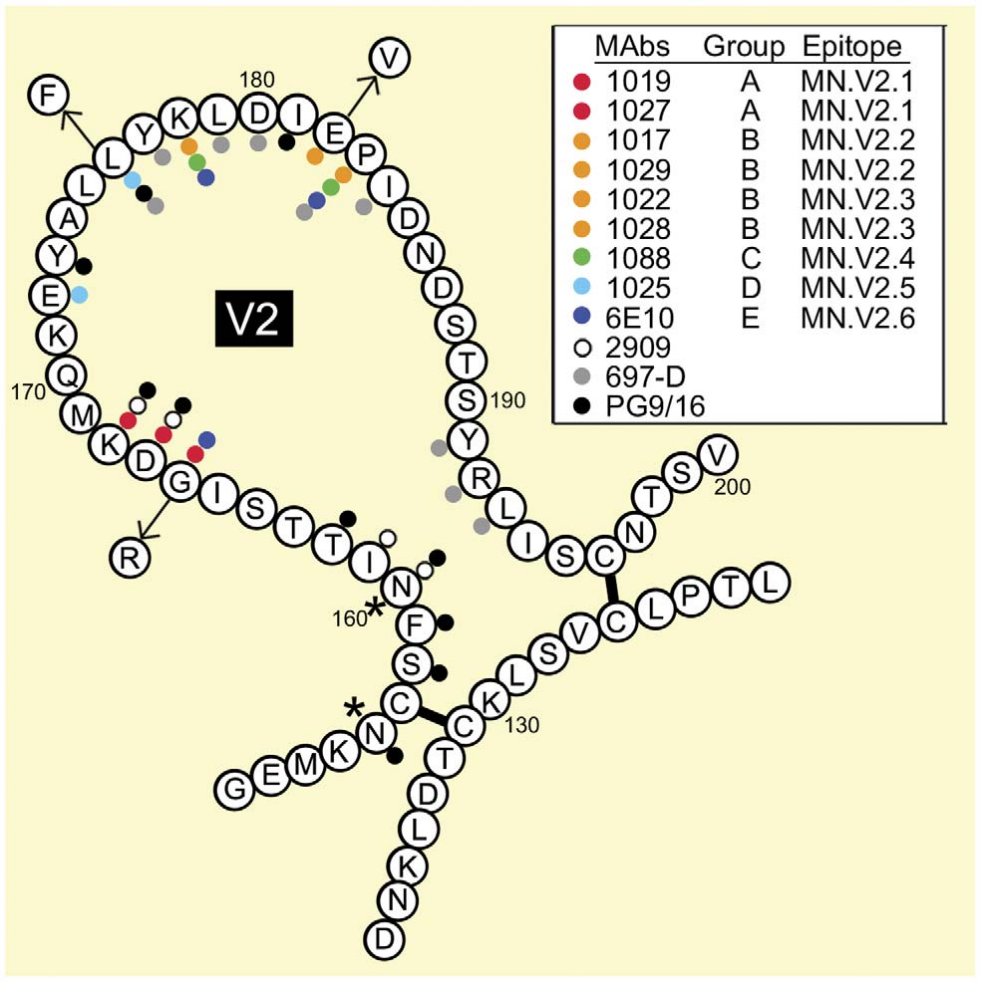
Location of amino acids critical for the binding of monoclonal antibodies to the V2 domain of gp120. A diagram of the gp120 V2 domain, incorporating amino acids from the MN_GNE_ strain of HIV-1, was created based on the disulfide structure of Leonard et al. [54]. The locations of the amino acids required for the binding of the MAbs to the V2 domain described in this paper, as well as 697-D [43], 2909 [47] and the potent PG9 and 16 [22] neutralizing antibodies are indicated by colored dots. Group, indicates competition group and epitopes from Table 4. Arrows, indicate the location of three radical amino acid polymorphisms found in MN-rgp120 compared to consensus clade B clinical isolates [60] and Keele [29]. Data for this figure was derived from previously published results [47], [22], [33].

Comparison of the MAbs revealed differences in cross-reactivity with envelopes from different strains, CD4 blocking activity, virus neutralizing activity, antibody competition, and the ability to block gp120 binding to α4β7. Site-directed mutagenesis identified eight positions in the V2 domain (166, 167, 168, 172, 176, 178, 182, and 183) that were critical for binding of one or more of the MAbs (Figure 6). These could be divided into two main clusters. One cluster involved residues 166–168, recognized by the 1019 and 1027 MAbs. These positions had previously been identified as being important for the binding of several potent neutralizing MAbs (e.g. PG9, 2909, CAP254) [32], [22], [20], [33], [21]. Five of the nine MAbs (1022, 1028, 1017, 1029, and 1088) recognized another cluster of amino acids (178–183) immediately adjacent to the α4β7 binding site [23]. One MAb (6E10) was unique in that it appeared to recognize amino acids from both clusters.

**Figure 7:**
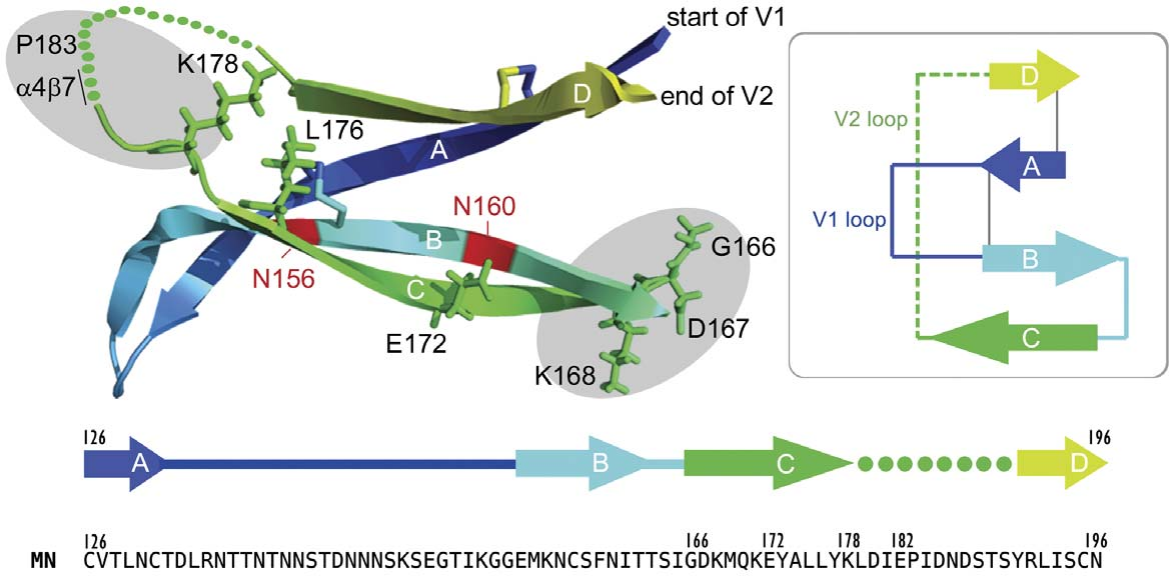
Location of epitopes recognized by monoclonal antibodies to the V2 domain of MN-rgp120. A diagram of the V1/V2 domains of gp120 taken from the 3U4E structure of McLellan et al. [34] is shown. The V1 and V2 domain include four anti-parallel strands (colored arrows) with a disordered loop region (dotted line) between the C and D strand. Light gray lines indicate disulfide bonds. The location of carbohydrate essential for the binding of the PG9 MAb is shown in red. The approximate locations of the two immunodominant clusters of residues critical for the binding of eight of the nine murine MAbs described in this study are indicated by shaded ovals. Thus the 166–168 cluster of amino acids occurs at the turn located at the B–C junction. The epitopes clustering in the region of residues 178–183 occur in the disordered peptide connecting the C and D strands on either side of D180, the central amino acid in the α4β7 binding site [23]. A linear diagram of the V2 domain and a listing of the sequence of the V2 domain of MN-rgp120 is provided at the bottom of the figure.

The structure of a scaffold comprising the V1 and V2 domains of gp120 bound to the broadly neutralizing PG9 MAb has recently been elucidated [34]. It was found (Figure 7) that the V1 and V2 domains consist of four anti-parallel β-sheets (A, B, C, and D). Interestingly the two immunodominant clusters of amino acids important for the binding of the mouse MAbs described in this study occurred at the opposite ends of the C strand and included residues from the turn at the B–C strand junction and the disordered peptide of the C–D connecting loop. The B–C turn includes positions 166, 167, and 168 that were important for the binding of the 1019 and 1027 murine MAbs described in this study (Figure 6) as well as for PG9, 2909 and several other potently neutralizing human MAbs [35], [34], [36]. The second cluster of epitopes overlaps the connecting loop between the C and D strands and is in close proximity to the α4β7 binding site (positions 179–181). Five of the nine MAbs described in this study (1022, 1028, 1017, 1029, and 1088) were found to recognize amino acids (residues 178–183) immediately adjacent to the α4β7 binding site.

Recent studies have shown that the central aspartic acid (D180) residue within the α4β7 recognition sequence (LDI/V) also appears to be an essential part of the conformational masking mechanism [37] used to shield neutralizing epitopes from neutralizing antibodies. Previous studies have referred to the V2 domain as the “global regulator of neutralization sensitivity” [18], [19] and have shown that the V2 domain interacts with other regions of gp120. Thus, the stem of the V2 domain is adjacent to the CD4 binding site [38], [39] and mutations in the V2 domain are known to affect CD4 binding as well as the binding of neutralizing antibodies that recognize both the V3 domain and the CD4 binding site [40], [41]. Astonishingly, deletion of the entire V2 domain can sometimes increase sensitivity to neutralization by antibodies to other regions while preserving virus infectivity [17], [19]. Finally, replacement of the V2 domain of a neutralization-resistant virus with the V2 domain of a neutralization-sensitive virus is able to confer the neutralization-sensitive phenotype [18].

Neutralizing MAbs targeting the V2 domain have been isolated from mice and rats immunized with recombinant envelope proteins, and from chimpanzees and humans infected with HIV-1 ([40]–[46], [32], [47], [33]. Until 2009, most of the neutralizing MAbs to the V2 domain were thought to be strain-specific, and less potent than antibodies to the V3 domain and CD4 binding site [48]. The recent isolation of several broadly neutralizing human MAbs to the V2 domain [22], [35], [49], as well as the correlation observed between protection and antibodies to the V2 domain in the RV144 trial [11], has rekindled interest in the V2 domain as a target for HIV-1 vaccine development. The prototype for broadly neutralizing antibodies to the V2 domain is the PG9 MAb, isolated from an elite neutralizer infected with a clade A virus [22]. The binding of this MAb appears to be dependent on sequences in both the V2 and V3 domains. Interestingly, binding by PG9 and other broadly neutralizing MAbs to the V2 domain is dependent on N-linked carbohydrate at positions 156 and 160 in the B strand, as well as a number of specific amino acids (e.g., residues 158, 159, 162, 167, 168, 173, 176, and 181) [22], [35], [34], [49]. Although the impact of glycosylation at these positions was not examined for the murine MAbs described in the study, both the MN- and IIIB-rgp120 immunogens used to elicit these MAbs possessed glycosylation sites at positions 156 and 160, and lacked a glycosylation site at position 162. The inability of PG9 to neutralize the MN strain of HIV-1 observed in these studies (Table 1) as well as its poor reactivity with MN-rgp120 by ELISA (Berman and Yu, unpublished results) may be due to differences in glycosylation at these positions.

Several studies have shown that neutralizing antibodies to the V2 domain are present in sera from HIV-infected humans. Walker et al. [20] reported that approximately 21% of the neutralizing activity in nineteen donors screened for high neutralization titers could be attributed to V2 MAbs with specificities similar to PG9 and PG16. Moore et al. [21] analyzed a potent neutralizing sera (CAP256) from a clade C infected individual and found much of the neutralizing activity was directed to the V2 domain at an epitope that overlapped, but was distinct from, the epitopes recognized by PG9 and PG16. This study reported that the binding of these antibodies was dependent on F159, N160, L165, K166, D167, and K169. The data from the present studies similarly show the importance of positions 166, 167, and 168 in the binding of MAbs from humans and mice. Although lysine (K) at position 169 was important for the binding of the sera from CAP256, the substitution of methionine (M) for valine (V) at position 169 in the V2 domain of the MN envelope protein had no effect on the binding of any of the MAbs in this study.

An important finding of this study was that four of the epitopes (MN.V2.2, MN.V2.3, MN.V2.4, and MN.V2.6) depended on positions 178, 182, and 183, adjacent to the highly conserved LDV/I sequon of the α4β7 binding site in the C–D connecting loop. The observation that the double mutation E182V-P183S blocked the binding of all of the MAbs described in this study (with the exception of 1025) suggests that these two residues are part of a structural element required for the functional conformation of the V2 domain. This result is in agreement with our previous observation [37] that the replacement of D with N at position 180 (HXB2 numbering) in the 108051 envelope protein induces a conformational change that markedly increases neutralization by inhibitors targeting the CD4 binding site in gp120 (e.g., CD4-IgG) and the MPER domain in gp41 (e.g., MAbs 2F5 and 4E10). The fact that positions 178, 182, and 183 are located on either side of the α4β7 recognition sequence suggests that MAbs to all four epitopes have the potential to block gp120 binding to α4β7 by steric interactions. If antibodies binding to these residues are found in sera from the RV144 trial, then non-neutralizing, α4β7-blocking antibodies may represent a possible correlate of protection. Studies are underway to analyze sera from the VAX003 and RV144 trials for antibodies of this type. In two previous Phase 3 HIV vaccine trials [50], [9], we reported that the AIDSVAX B/B and AIDSVAX B/E vaccines containing MN-rgp120 elicited robust immune responses to linear epitopes in the V2 domain, as measured by binding to synthetic peptides from the MN and A244 strains of HIV-1. Both peptides span the regions that contain the epitopes recognized by the MAbs described in this paper, but all except 1088 fail to bind to these.

Given the fact that MN-rgp120 is able to stimulate the formation of conformation-dependent antibodies to the V2 domain, it is surprising that we didn't observe greater cross-reactivity and greater neutralizing activity. Several possible explanations may account for this. First, there were several radical amino acid polymorphisms in the MN-rgp120 immunogen that are uncommon in clade B isolates (Figure 4). These include glycine (G) rather than arginine (R) at position 166, leucine (L) rather than phenylalanine (F) at position 176, and glutamic acid (E) rather than valine (V) at position 182. These may account for the strain specific anti-V2 response. Second, it is possible that the monomeric immunogens used in the AIDSVAX vaccines were unable to replicate conformational epitopes formed by quaternary interactions in the gp160 homotrimer, essential for the production of some neutralizing antibodies to the V2 domain. Finally it is possible that the immune response to the V2 domain elicited in humans differs from that elicited in mice with respect to formation of antibodies targeting carbohydrate-containing epitopes such as PG9.

The results described in this paper add to our understanding of the specificity of the immune response to the V2 domain of gp120. They show that the antigenic structure of the V2 domain is extraordinarily complex, containing at least eight to ten conformation-dependent epitopes including the six described in this paper along with those recognized by the PG9, PG16, 2909, C108g, and 10/76b MAbs [46], [51], [47], [22] and multiple conformation-independent epitopes. The studies described show that antibodies to the V2 domain can inhibit the binding of MN-rgp120 binding to α4β7 and highlight epitopes in the V2 domain that deserve further investigation. This is particularly significant in the context of the protection observed in the RV144 clinical trial, compared to the lack of protection observed in the VAX003 and VAX004 clinical trials. Such studies will help clarify the extent to which the immune response to the V2 domain elicited by vaccine antigens replicates the immune response to natural infection, and may provide insights useful for the validation of antibodies to the V2 domain as the factor responsible for protection in the RV144 trial.

## Materials and Methods

### gp120 sequences and nomenclature

The standard single letter code is used to designate the amino acids. The numbering of amino acids in gp120 is provided with reference to the standard HXB2 numbering system. The sequence of the MN isolate incorporated in the MN-rgp120 immunogen (MN_GNE_) has been described elsewhere (GSID HIV Data Browser, http://www.gsid.org/gsid_hiv_data_browser.html). The gp120 MN_GNE_ is two percent different than the original MN gp120 sequence published by Gurgo et al. [52]. The sequence of IIIB reported in this paper was described by Muesing et al. [53]. All the conserved and variable domain assignments were based on the 2D structure of gp120 [54].

### HIV envelope proteins

The HIV envelope proteins including MN-rgp120 were produced as described previously [1]–[3]. Purified MN-rgp120 protein was biotinylated by amine-coupling using the EZ-Link™ NHS Biotin kit (Pierce) following the manufacturer instructions. A 20-fold molar excess of EZ-Link™ NHS Biotin reacted with MN-rgp120 protein for 30 min at room temperature. The biotinylated protein was then dialyzed against PBS to remove non-reacted biotin. Purified MN-rgp120 was labeled with ^125^I as described previously.

### Monoclonal antibodies and immunoassays

Mouse monoclonal antibodies reactive with the V2 domain of MN-rgp120 were identified from a collection of thirty-six monoclonal antibodies produced in the mid-1990s at Genentech, Inc. (S. San Francisco, CA) [27], [28] and purified using Protein A Sepharose (Pharmacia, Piscataway, NJ). MAbs were conjugated to horseradish peroxidase (HRPO) according to the method of Nakane et al. [55]. The cross-reactivity of the MAbs to recombinant gp120s from clade B (IIIB, NY5, JRCSF, and MN), clade A (Z321), clade D (Z6), and clade E (A244) isolates of HIV-1 was determined by ELISA, as described previously [27], [28]. The ability of the MAbs to inhibit the binding of [^125^I]-labeled MN-rgp120 to cell surface CD4 was measured as described previously [28]. The binding of MAbs to chemically denatured MN-rgp120 was determined by ELISA using reduced and carboxymethylated gp120 [54]. MAbs to α4β7 integrins, including the rat anti-β7 MAbs FIB21, FIB22, FIB30, FIB504, LS722, and the anti-α4β7 mouse MAb, Act-1 [56] were a gift from Eugene C. Butcher (Stanford University School of Medicine, Stanford, CA).

### Site-directed mutagenesis and expression of MN-rgp120 V2 mutants

Site-directed mutagenesis of regions in the MN-gp120 V2 region was performed using the method of Kunkel et al. [57]. The pRKMN120 plasmid encoding the entire envelope protein of the MN-gp120 was transformed into the dut^−^ung^−^ CJ236 *E. coli* strain and then grown in the presence of MK107 helper phage for the purpose of generating a single stranded DNA template. 33- to 40-mer oligonucleotides incorporating the desired mutation were utilized to insert the mutations into the V2 region. The mutants were transformed into bacteria and the colonies were screened by sequencing (Sequenase 2.0, U.S. Biochemicals) to check for the correct mutations. These plasmids containing the MN gp120 V2 mutants were then transfected into the 293 human embryonic adenocarcinoma cell line using the calcium phosphate method described by Graham and van der Eb [58]. After three days, secreted rgp120 containing the V2 mutations was collected and quantitated using a gp120 ELISA.

### Expression of MN envelope fragments

Fragments of MN-rgp120 were expressed in mammalian cells as described previously [28]. Briefly, selected fragments of MN-rgp120 gene were generated by PCR and fused, in frame, at the 5′ end to a fragment of the herpes simplex virus type 1 glycoprotein D (gD-1) gene (containing the signal sequence and 27 amino acids of the mature gD-1 protein) and at the 3′ end to translational stop codons. A 3′ Xho1 site engineered into the gD-1 fragment allowed for in-frame ligation of PCR-generated gp120 fragments. The chimeric fragments were cloned into the pRK5 mammalian cell expression vector containing the CMV promoter and enhancer elements and an SV40 polyadenylation site. Calcium phosphate-precipitated plasmid DNA encoding the chimeric gp120 fragments was transfected in the embryonic kidney adenocarcinoma-derived 293 cell line (Invitrogen, Carlsbad, CA), and growth conditioned cell culture medium was collected for immunoassays (described below).

### Localization of MAb binding sites using fragments of the MN-rgp120

The ability of MAbs to bind to various fragments of MN-rgp120 was determined by ELISA. Three days after transfection, the 293 cell culture supernatants were harvested and the gp120 fragments were captured onto wells of microtiter plates coated with a murine MAb (5B6) specific for the gD-1 flag epitope. The ability of HRPO-conjugated MAbs to bind to the captured fragments was measured by ELISA. The cell culture supernatants containing the MN-rgp120 fragments were then added to the wells and incubated for 2 hr at room temperature. The plates were washed twice with PBT (Phosphate-buffered saline with 0.05% Tween-20) and then incubated for 2 hr at room temperature with a pre-titrated amount of HRPO-labeled MAb. Plates were washed again in PBT buffer and then developed with o-phenylenediamine tablets (Sigma, St. Louis, MO) for 15 minutes. The reaction was stopped by the addition of 2.5M H_2_SO_4_. The plates were read in a microtiter plate spectrophotometer at a wavelength of 492 nm.

### Binding of V2 MAbs to MN-rgp120 V2 mutants

A capture ELISA similar to that described previously [59] was used to measure the binding of MAbs to MN-rgp120 variants mutated in various domains. Briefly, microtiter assay plates were coated (5 µg/mL in PBS, overnight at 4°C) with an affinity purified polyclonal sheep antisera (D7234, International Enzymes, Fallbrook, CA) to a sequence in the C terminus of gp120. The plates were blocked with PBS containing 0.7% bovine serum albumin for 1 hr at room temperature. Equivalent amounts of the tissue culture supernatants from the transiently expressed gp160 mutants were added and incubated for 2 hr at 37°C followed by washing 3 times in PBT buffer. Various MAbs (0.16 mg in 100 µl), diluted in a PBS BSA buffer, were added to each well and incubated for 1.5 hours at 37°C. The plates were then washed in PBT buffer and then incubated with HRPO-conjugated goat anti-mouse IgG (1/2000 dilution in PBT) for 1 hr at room temperature. The plates were developed with OPD and stopped by the addition of 2.5N H_2_SO_4_.

### MN-rgp120 CD4 blocking assays

The CD4 blocking assay was described elsewhere [27], [28]. Briefly, Chinese hamster ovary cells continuously expressing full length human CD4 were plated in 96 well dishes until 90% confluent. [^125^I]-labeled MN-rgp120 (2 nM) was mixed with serially diluted MAbs and then transferred onto the cells. The assay was performed in a 50∶50 mix of F12/DMEM (Gibco, Grand Island, NY) containing 10% fetal bovine serum, 0.01% NaAzide and 12 mM HEPES. The mixture was allowed to react with the cells for 2 hr, and then washed four times with assay medium. The washed cells were then solubilized in 100 µL of 1.0 N NaOH for 30 min and 90 µL was removed for counting in a gamma counter. The percentage of inhibition was calculated by the equation 100-[100*(experimental – background)/(antibody control – background)].

### Virus neutralization assays

A colorimetric MT-2 syncytia inhibition assay, similar to that described previously [27], [28] was initially used to screen MAb supernatants for neutralizing activity. For MAb characterization, a standard TZM-bl neutralization assay was used to assess the ability of V2 MAbs to neutralize pseudotyped virus from the MN-3 [10]. IC50 curves were calculated from serial 3-fold dilutions from 1/20–1/43,740 in TZM-bl cells. The TZM-bl cells and the pSG3^Δenv^ expression plasmid were obtained from the NIH AIDS Research & Reference Reagent Program (contributed by Drs. John C. Kappes & Xiaoyun Wu). The MN-3 envelope expression plasmid was a gift from Dr. David Montefiori. For standardization of protocols, we used HIV-1 MN-3 pSG^Δenv^ 293T/17 pseudovirus, a generous gift from Jochen Schmidt and Hagen Von Briesen at the HIV Specimen Cryorepository, Fraunhofer Insitute (IBMT), Sulzbach, Germany.

### α4β7 binding assays

Purified MN-rgp120 protein was biotinylated as described above. The binding of biotinylated MN-rgp120 to α4β7 was carried out using the α4β7^+^, β1^−^ RPMI-8866 B lymphoma cell line [30], [31] kindly provided by Eugene C. Butcher (Stanford University School of Medicine, Stanford, CA). Cells were grown in RPMI-1640 supplemented with 10% FBS, L-glutamine, sodium pyruvate, penicillin-streptomycin, non-essential amino acids and 2-mercaptoethanol. The binding of MN-rgp120 to α4β7 was measured by flow cytometry. For this assay, Fc receptors on the RPMI-8866 cells were first blocked by pre-treatment with rabbit IgG. The cells were then incubated for 1 hr at 4°C with biotinylated MN-rgp120 in staining buffer (0.5% BSA, 10 mM HEPES, 150 mM NaCl, 100 mM CaCl_2_ and 1 mM MnCl_2_). APC-conjugated streptavidin (BioLegend, CA) was used to detect biotinylated gp120 binding to the cells. For blocking experiments, MN-rgp120 (20 µg/ml) was pre-incubated with the V2 MAbs (10 µg/ml) for 1 hr at room temperature and then added to the cells. For blocking experiments with the integrin MAbs, cells were pre-incubated with the integrin MAbs (10 µg/ml) for 30 min at 4°C, washed twice with 0.5% BSA in PBS and then the biotinylated MN-rgp120 was added. Data were collected with a FACSAria (Becton-Dickinson Biosciences, San Jose, CA) at the UC Santa Cruz Flow Cytometry Facility and analyzed with FlowJo 8.8.6. software (TreeStar).

### Statistical analysis

Differences between the ability of MN-rgp120 to bind α4β7 in the absence or presence of V2 antibodies were analyzed with one way ANOVA with Bonferroni correction, using Prism 5.0 (GraphPad Software). *P* values of <0.05 were considered to be statistically significant.

## Supporting Information

Figure S1 **Inhibition of rgp120 binding to CHO cell lines expressing CD4 by monoclonal antibodies to the V2 domain of gp120.** CHO cells expressing membrane-bound human CD4 were plated in 96-well microtiter plates until they were 90% confluent. Serial dilutions of MAbs were pre-incubated with a fixed amount of [^125^I]-gp120 (2 nM) and then incubated with the cells for 2 hr. The cells were then washed four times and the cells solubilized in 100 µL of 0.1 N NaOH. Specific binding was calculated as described previously [27]. MAb 1024, a CD4-blocking antibody directed to the C4 domain of gp120, served as a positive control.
(PDF)
