# Supplementary material for: Monoclonal Antibodies to the V2 Domain of MN-rgp120: Fine Mapping of Epitopes and Inhibition of α4β7 Binding
Source: PLoS One. 2012 Jun 13;7(6):e39045. doi: 10.1371/journal.pone.0039045 (PMC3374778; doi:10.1371/journal.pone.0039045)
Supplement: Figure S1 — Inhibition of rgp120 binding to CHO cell lines expressing CD4 by monoclonal antibodies to the V2 domain of gp120. CHO cells expressing membrane-bound human CD4 were plated in 96-well microtiter plates until they were 90% confluent. Serial dilutions of MAbs were pre-incubated with a fixed amount of [125I]-gp120 (2 nM) and then incubated with the cells for 2 hr. The cells were then washed four times and the cells solubilized in 100 µL of 0.1 N NaOH. Specific binding was calculated as described previously [27]. MAb 1024, a CD4-blocking antibody directed to the C4 domain of gp120, served as a positive control. (PDF) [file pone.0039045.s001.pdf]

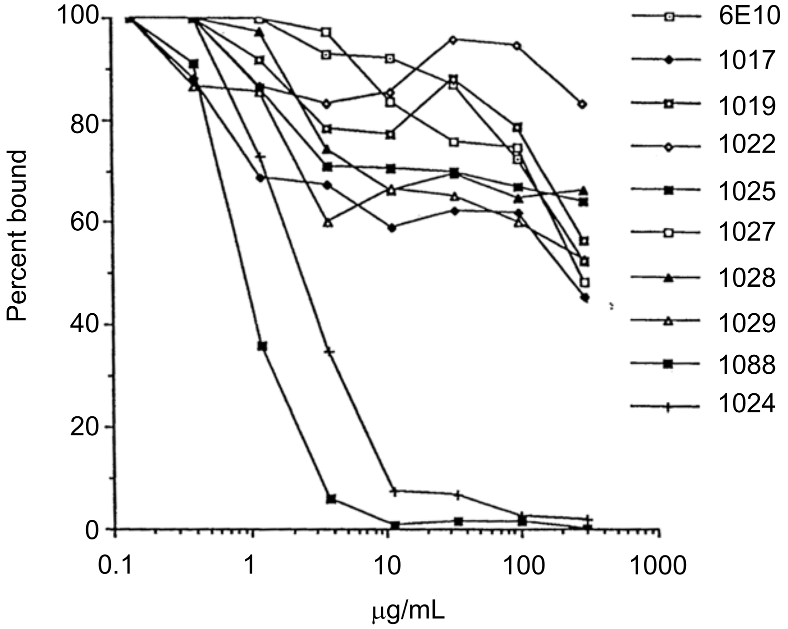

Supplemental Figure S1. Inhibition of rgp120 binding to CHO cell lines expressing CD4 by monoclonal antibodies to the V2 domain of gp120. CHO cells expressing membrane-bound human CD4 were plated in 96-well microtiter plates until they were 90% confluent. Serial dilutions of MAbs were pre-incubated with a fixed amount of [^{125}I]-gp120 (2 nM) and then incubated with the cells for 2 hr. The cells were then washed four times and the cells solubilized in 100 µL of 0.1 N NaOH. Specific binding was calculated as described previously. MAb 1024, a CD4-blocking antibody directed to the C4 domain of gp120, served as a positive control.
